# Impact of preoperative biliary drainage on outcomes of pancreaticoduodenectomy in severe hyperbilirubinemia

**DOI:** 10.1007/s00464-025-12027-2

**Published:** 2025-08-13

**Authors:** Fahim Kanani, Nir Messer, Bar Cohen, Ella Falk, Yaacov Goykhman, Nir lubezky

**Affiliations:** 1 Department of HPB and Transplant Surgery, Division of Surgery, Gray Faculty of Medical and Health Sciences, Tel Aviv University, Tel Aviv Medical Center 6 Weizmann Street, 6423906 Tel Aviv, Israel; 2 Department of Surgery, Gray Faculty of Medical and Health Sciences, Wolfson Medical Center, Tel Aviv University, Tel Aviv, Israel; 3 Gray Faculty of Medical and Health Sciences, Tel Aviv University, Tel Aviv, Israel

**Keywords:** Pancreaticoduodenectomy, Hyperbilirubinemia, Preoperative biliary drainage, Postoperative mortality, Surgical outcomes, Jaundice, Pancreatic cancer

## Abstract

**Background:**

Optimal management of patients with severe hyperbilirubinemia (> 14.6 mg/dL) undergoing pancreaticoduodenectomy remains controversial.

**Methods:**

Single-center retrospective study of 665 pancreaticoduodenectomies (2007–2024). Patients were stratified: no preoperative biliary drainage (PBD) with high (> 14.6mg/dL, *n* = 83) or low (< 14.6mg/dL, *n* = 312) bilirubin; PBD for low bilirubin (*n* = 140); PBD for high bilirubin (*n* = 113). Primary outcomes: 90-day mortality, morbidity, and specific complications.

**Results:**

Ninety-day mortality was significantly higher in non-PBD patients with high bilirubin (13.3%) compared to other groups (2.9–5.7%, *p* = 0.001). Overall morbidity was also higher in this group (29.0% vs. 12.8–20.7%, *p* < 0.001). While PBD groups showed higher surgical site infections (21.2–26.4% vs. 9.6%, *p* = 0.001), non-PBD high-bilirubin patients demonstrated increased ARDS (6.0% vs. 0–1.4%, *p* = 0.016) and reoperation rates (18.1% vs. 9.3–11.5%, *p* = 0.029). Multivariate analysis identified preoperative bilirubi*n* > 14.6mg/dL as an independent predictor of 90-day mortality.

**Conclusion:**

Preoperative bilirubin ≥ 14.6mg/dL independently predicts perioperative morbidity and mortality following pancreaticoduodenectomy. These findings support preoperative biliary drainage in patients with severe hyperbilirubinemia to optimize surgical outcomes, despite PBD-associated infectious complications.

**Supplementary Information:**

The online version contains supplementary material available at 10.1007/s00464-025-12027-2.

Pancreatic ductal adenocarcinoma (PDAC) is a highly lethal cancer, with a 5-year survival rate below 10% [1, 2].Obstructive jaundice, affecting 70–80% of patients at presentation, is a significant perioperative risk factor [3, 4]. PBD, via endoscopic retrograde cholangiopancreatography (ERCP) or percutaneous transhepatic drainage (PTD), is used to manage jaundice before pancreaticoduodenectomy, however these interventions carry notable risks which may delay surgery or even increase perioperative infectious complications due to bile contamination [5]. Guidelines recommend PBD in cases requiring neoadjuvant therapy, cholangitis, severe nutritional deficits, or delayed surgery [6, 7].

The optimal management of patients with severe hyperbilirubinemia (serum bilirubi*n* > 14.6 mg/dL) remains debated. Van der Gaag et al.’s randomized controlled trial showed increased complications with preoperative biliary drainage, though their study excluded patients with bilirubin levels above 14.6 mg/dL [1]. While subsequent studies have presented conflicting evidence, current data suggests that clinical status, rather than absolute bilirubin levels alone, may better guide treatment decisions [8–10].

An uncommon dilemma clinician often face is whether to operate or biliary drain patients diagnosed with periampullary cancer that present with marginally high bilirubin and following a rapid preparation for surgery, bilirubin exceeds 14.6 mg/dL, without signs of sepsis or malnutrition.

Given the ongoing controversy and the limited literature regarding the management of patients with bilirubi*n* > 14.6 mg/dL, we analyzed our database of pancreatic surgeries to investigate our perioperative outcomes in patients with elevated bilirubin who underwent pancreaticoduodenectomy without preoperative-drainage [11, 12].

## Methods

We included patients over 18 years who underwent Whipple’s procedure for periampullary cancer at Tel Aviv Medical Center from January 2007 to September 2024. Out of 1,450 pancreatic surgeries reviewed, 805 underwent the Whipple procedure, 17 were removed due to non-valid data; 788 met the inclusion criteria, due to missing critical data about preoperative bilirubin level or preoperative PBD status, we removed other 123 records leaving 665 valid cases.

Cholestasis was defined as a total bilirubin level > 2.5mg/dL. Tumors were staged according to the eighth edition of the TNM cancer staging manual of the American Joint Committee on Cancer [12].

Data collection was performed through our prospectively maintained pancreatic surgery database. All pancreaticoduodenectomies were performed by a dedicated team of 4 fellowship-trained HPB surgeons, each with > 5 years post-fellowship experience from high-volume centers (Mount Sinai, UPMC). Cases were distributed equally (22–28% per surgeon) following standardized protocols to minimize inter-operator variability. Our institution maintains high-volume status (> 40 PDs/year), increasing from 40–50 to 55–70 cases annually during the study period..This database is updated daily and includes comprehensive data from multidisciplinary team meetings with oncologists and gastroenterologists, ensuring optimal interpretation of clinical, radiological, and pathological findings. We systematically collected patient demographics, American Society of Anesthesiologists (ASA) physical status classification, and comprehensive laboratory values including total and direct bilirubin, liver function tests, albumin, complete blood count, and coagulation profiles. All laboratory values were obtained within one week before surgery. Radiological staging and tumor characteristics were documented based on preoperative computed tomography or magnetic resonance imaging. When TNM staging and/or resectability were unclear based on CT scan reports and multidisciplinary meeting discussions, an expert radiologist conducted an additional review.

From our surgical database, we extracted operative variables including procedure duration, estimated blood loss, transfusion requirements, type of reconstruction, and vascular resection needs. Postoperative outcomes were tracked through our institutional complications registry using the Clavien-Dindo classification system. We documented specific complications using standardized international criteria: pancreatic fistula (per International Study Group of Pancreatic Surgery definition) [13], delayed gastric emptying, post-pancreatectomy hemorrhage, intra-abdominal abscess formation, surgical site infections, acute respiratory distress syndrome, and pneumonia. Perioperative antibiotic prophylaxis consisted of broad-spectrum coverage (piperacillin-tazobactam or equivalent) initiated 24 h before ERCP and continued for 48–72 h post-procedure based on clinical assessment. Outcome measures included 30-, 60-, and 90-day mortality, length of hospital stay, reoperation needs, and readmission rates. We selected 90-day mortality as our primary endpoint based on evidence that pancreaticoduodenectomy-related deaths frequently occur beyond 30 days, with 90-day mortality often doubling 30-day rates and better capturing procedure-related complications. Based on established literature thresholds [14–17], The 14.6 mg/dL threshold was selected based on Van der Gaag et al.’s landmark randomized controlled trial [1] which excluded patients above this level due to safety concerns. This cutoff has been subsequently validated as the definition of severe hyperbilirubinemia in multiple studies examining perioperative outcomes [11, 12, 23], This threshold represents the point above which the physiological derangements of hyperbilirubinemia significantly impact surgical outcomes, making it clinically relevant for decision-making regarding preoperative biliary drainage. Patients were categorized into four groups based on PBD status and bilirubin levels: No PBD-High (No PBD, preoperative bilirubi*n* > 14.6mg/dL, *n* = 83), No PBD-Low (No PBD, preoperative bilirubi*n* < 14.6 mg/dL, *n* = 312), PBD-Low (PBD, initial bilirubi*n* < 14.6 mg/dL, *n* = 140), PBD-Reduced (PBD, initial bilirubi*n* > 14.6 mg/dL reduced to < 14.6mg/dL preoperatively, *n* = 113); Low bilirubin patients (< 14.6 mg/dL) were included to: validate our surgical outcomes against international benchmarks; confirm expected PBD-related complications; isolate hyperbilirubinemia effects from PBD effects; provide statistical power for multivariate analysis; and establish a clinical decision-making framework for rising bilirubin levels during preoperative preparation; we excluded PBD-High (PBD, initial and preoperative bilirubi*n* > 14.6 mg/dL, *n* = 17) due to champion’s small-size to avoid potential bias. The groups were compared for pre-operative, intraoperative and postoperative outcomes and for mortality in 90 days.

Statistical analysis was performed using SPSS Statistics version 27.0 (IBM Corp., Armonk, NY). Continuous variables were presented as mean ± standard deviation or median with interquartile range based on their distribution, with normality assessed using the Shapiro–Wilk test. Categorical variables were expressed as frequencies and percentages. Between-group comparisons were conducted using Student’s t-test or Mann–Whitney U test for continuous variables and chi-square or Fisher’s exact test for categorical variables as appropriate. Multivariate logistic regression analysis was performed to identify independent predictors of major complications and mortality, with *p* < 0.05 considered statistically significant.

This study was conducted in accordance with the ethical standards of the institutional research committee of Tel Aviv Medical Center and with the 1964 Helsinki Declaration and its later amendments. The requirement for informed consent was waived by the ethics committee due to the retrospective nature of the study.

## Results

Patient demographics and clinical characteristics demonstrated distinct patterns across treatment groups (Table 1). Among 665 evaluable patients who underwent pancreaticoduodenectomy, 395 (59.4%) did not receive preoperative biliary drainage (PBD), while 270 (40.6%) underwent PBD. Non-PBD patients were stratified by bilirubin level (high > 14.6mg/dL, *n* = 83; low < 14.6mg/dL, *n* = 312), while PBD patients were categorized by pre-drainage bilirubin and post-drainage response (initially low, *n* = 140 and initially high with reduction, *n* = 113). Age distribution showed marginal variation (*p* = 0.074), with high-bilirubin PBD patients demonstrating the highest median age (74.65 ± 5.3 years) compared to low-bilirubin non-PBD patients (64.12 ± 5.2 years). Serum albumin levels differed significantly across groups (*p* < 0.001), with lowest values observed in PBD patients with reduced bilirubin (35.7 ± 5.2 g/L) versus low-bilirubin non-PBD patients (40.2 ± 4.8 g/L). Gender distribution demonstrated significant variation (*p* = 0.011), with male predominance in high-bilirubin non-PBD (66.3%), PBD with reduced bilirubin (67.3%), and high-bilirubin PBD (70.6%) groups. Clinical jaundice was significantly more prevalent in high-bilirubin groups regardless of drainage status (88.2–99.0%, *p* < 0.001). Preoperative tissue diagnosis rates varied significantly (*p* < 0.001), being highest in low-bilirubin PBD patients (82.5%) and lowest in high-bilirubin non-PBD patients (30.5%). Comorbidity profiles remained comparable across all groups, including diabetes mellitus (26.7–46.4%, *p* = 0.178), hypertension (43.6–60.0%, *p* = 0.490), and cardiac disease (14.1–25.0%, *p* = 0.615).

**Table 1:** Patient demographics and clinical characteristics by preoperative biliary status

Characteristic	No PBD, High Bilirubin (*n* = 83)	No PBD, Low Bilirubin (*n* = 312)	PBD Group (*n* = 253)	*p*-value
Demographics				
Age, years	69.5 (65.2–73.8)	64.1 (59.4–68.9)	67.5 (63.0–72.1)	0.074
Male sex	55 (66.3)	166 (53.2)	147 (58.1)	0.011*
BMI, kg/m^2^	26.2 (24.1–28.2)	25.3 (23.1–27.6)	25.4 (23.3–27.5)	0.412
Albumin, g/L	38.8 (35.6–42.0)	40.2 (36.8–43.6)	37.3 (34.1–40.5)	< 0.001**
Clinical parameters				
Preoperative bilirubin, mg/dL	17.4 (14.6–27.7)	4.1885 (0.13 -14.5)	2.6 (0.17–12.6)	< 0.001**
Neoadjuvant chemotherapy	0 (0.0)	42 (14.0)	28 (13.0)	0.009**
Comorbidities				
Diabetes mellitus	25 (30.9)	114 (37.6)	98 (40.3)	0.178
Hypertension	34 (43.6)	142 (48.0)	120 (52.0)	0.490
Smoking history	27 (32.5)	116 (37.2)	106 (41.9)	0.100

Data are presented as median (interquartile range) for continuous variables and n (%) for categorical variables. High bilirubin defined as > 14.6 mg/dL; low bilirubin defined as < 14.6 mg/dL

*PBD* preoperative biliary drainage, *BMI* body mass index

Pathological and operative characteristics exhibited distinct patterns across treatment groups (Table 2). Ductal adenocarcinoma predominated in high-bilirubin non-PBD patients (57.8%) compared to low-bilirubin non-PBD patients (14.1%) and PBD patients (33.6%) (*p* < 0.05). The low-bilirubin non-PBD group consisted primarily of non-PDAC pathologies (85.9%), including ampullary adenocarcinomas, neuroendocrine tumors, and distal bile duct tumors. Pancreatic head tumors were significantly more common in high-bilirubin non-PBD patients (60.2%) versus low-bilirubin non-PBD (16.3%) and PBD groups (32.4%) (*p* < 0.05). Tumors ≥ 2cm constituted the majority of cases across all cohorts (84.2–92.6%, *p* = 0.039). Regional lymph node metastasis was present in 33.7% of high-bilirubin non-PBD cases compared to 14.4% in low-bilirubin non-PBD and 39.9% in PBD patients (*p* < 0.05). Perineural invasion (49.4–54.2%) and lymph-vascular invasion (37.0–51.0%) showed no significant differences between groups (*p* > 0.05). Stage ≤ IIB disease was markedly more common in high-bilirubin non-PBD (75.8%) and PBD patients (71.6%) compared to low-bilirubin non-PBD patients (29.7%) (*p* < 0.05). Operative characteristics revealed significant differences in surgical approach, with open procedures predominating in high-bilirubin non-PBD patients (57.8%) compared to low-bilirubin non-PBD (18.6%) and PBD (32.0%) groups (*p* < 0.001). Prolonged operative time (> 6 h) was almost universal in low-bilirubin non-PBD (98.1%) and PBD patients (96.1%) but less frequent in high-bilirubin non-PBD patients (84.9%) (*p* < 0.001). Intraoperative blood loss varied significantly (*p* < 0.001), with median values from 172mL (IQR:50–300) in No PBD-High to 380mL (IQR:150–550) in PBD-High. Vascular resection was performed in 5.9–31.9% of cases (*p* > 0.05). Main pancreatic duct diameter data was available for 68% of patients. Median duct diameter was 7 ± 0.8 mm in high-bilirubin non-PBD, 5.6 ± 0.9 mm in low-bilirubin non-PBD, 5.1 ± 0.8 mm in PBD-low bilirubin, and 5.5 ± 0.8 mm in PBD-reduced groups (*p* > 0.05).

**Table 2:** Pathological and operative characteristics by preoperative biliary status (*n* = 570)

Characteristic	No PBD, High Bilirubin (*n* = 83)	No PBD, Low Bilirubin (*n* = 312)	PBD Group (*n* = 253)	Total	*p*-value
Tumor Size					0.039*
< 2 cm	9 (10.8)	23 (7.4)	40 (15.8)	72 (12.6)	
≥ 2 cm	74 (89.2)	289 (92.6)	213 (84.2)	576 (87.4)	
Histopathology					< 0.05*
Ductal adenocarcinoma	48 (57.8)	44 (14.1)	85 (33.6)	177 (31.1)	
Other	35 (42.2)	268 (85.9)	168 (66.4)	471 (68.9)	
Pathological staging factors					
Perineural invasion	41 (49.4)	162 (52.0)	137 (54.2)	340 (52.6)	> 0.05
Lymphovascular invasion	31 (37.0)	125 (40.0)	129 (51.0)	285 (44.2)	> 0.05
Regional lymph node metastasis	28 (33.7)	45 (14.4)	101 (39.9)	174 (27.0)	< 0.05*
AJCC 8th Edition stage					< 0.05*
Stage ≤ IIB	63 (75.8)	93 (29.7)	181 (71.6)	337 (56.0)	
Others	20 (24.2)	219 (70.3)	72 (28.4)	311 (44.0)	
Operative parameters					
Operative time > 6 h	45/53 (84.9)	256/261 (98.1)	98/102 (96.1)	399/416 (95.9)	< 0.001***
Operative outcomes					
Blood loss > 500 mL	11/24 (45.8)	44/98 (44.9)	29/29 (100)	84/151 (55.6)	0.001**
Transfusion required	29/35 (82.9)	179/184 (97.3)	54/54 (100)	262/273 (96.0)	< 0.001***

Data are presented as n (%) or fraction (%). High bilirubin defined as > 14.6 mg/dL; low bilirubin defined as < 14.6 mg/dL. Staging according to AJCC Cancer Staging Manual, 8th Edition. Stage ≤ IIB includes T1-3N0M0 and T1-3N1M0; Others includes not fully classifiable cases. PBD = preoperative biliary drainage. Valid data available for 570 cases out of 655 (missing data in 85 cases, 13.0%)

^*^p < 0.05, **p < 0.01, ***p < 0.001

Postoperative complications exhibited distinct patterns across treatment groups (Table 3). The PBD cohort demonstrated significantly higher rates of infectious complications, including surgical site infections (24.2% vs. 9.6%, *p* = 0.001) and abdominal abscesses (15.1% vs. 7.2%, *p* = 0.028) compared to non-drained patients with high bilirubin. Conversely, high-bilirubin non-PBD patients experienced increased systemic complications, with higher rates of pneumonia (8.4% vs. 2.4%, *p* = 0.087) and ARDS (6.0% vs. 0.8%, *p* = 0.016). Anastomotic complications varied significantly, with bile leaks occurring in 1.2–4.0% across groups, highest in the PBD cohort. Gastrointestinal anastomotic leak distribution was particularly striking, with significantly lower rates in high-bilirubin non-PBD patients (3.6%) compared to low-bilirubin non-PBD (4.5%) and PBD groups (4.3%) (*p* = 0.941). Similarly, pancreatic fistula rates were markedly higher in high-bilirubin non-PBD patients (32.5%) versus low-bilirubin non-PBD (23.1%) and PBD (27.3%) groups (*p* = 0.178), with Grade C fistulas showing significant variation (*p* < 0.001). The need for reintervention was significantly higher in high-bilirubin non-PBD patients, with 18.1% requiring return to the operating room versus 10.3% in the PBD group (*p* = 0.029). Ultimately, severe complications were significantly more prevalent in jaundiced patients regardless of drainage status (39.8% in Group 1 vs. 33.3% in Group 2, with Group 3 at 40.7%, *p* = 0.002).

**Table 3:** Post-operative complications by group

Complication	No PBD, High Bilirubin (*n* = 83)	No PBD, Low Bilirubin (*n* = 312)	PBD Group (*n* = 253)	*p*-value
Surgical site complications				
Delayed gastric emptying	15 (18.1%)	58 (18.6%)	31 (12.3%)	0.139
Surgical site infection	8 (9.6%)	40 (12.8%)	61 (24.2%)	**0.001†**
Anastomotic complications				
GI anastomotic leak	1 (1.2%)	2 (0.65%)	2 (0.79%)	0.941
Bile leak	1 (1.2%)	4 (1.2%)	7 (2.8%)	0.188
Pancreatic fistula	27 (32.5%)	72 (23.1%)	71 (28.0%)	0.178
– Grade A	13 (15.7%)	35 (11.2%)	28 (11.1%)	0.490
– Grade B	7 (8.4%)	34 (10.9%)	37 (14.6%)	0.339
– Grade C	7 (8.4%)	3 (1.0%)	5 (2.0%)	** < 0.001**
Delayed postoperative bleeding	4 (4.8%)	8 (2.6%)	12 (4.7%)	0.334
Abdominal complications				
Peritonitis	4 (4.8%)	5 (1.6%)	10 (4.0%)	0.326
Abdominal abscess	6 (7.2%)	27 (8.7%)	38 (15.1%)	0.028*
POVH/Dehiscence	4 (4.8%)	11 (3.5%)	15 (6.0%)	0.436
Systemic complications				
Pneumonia	7 (8.4%)	17 (5.4%)	6 (2.4%)	0.087*
ARDS	5 (6.0%)	4 (1.3%)	2 (0.8%)	0.016*
MI/AF	4 (4.8%)	8 (2.6%)	12 (4.8%)	0.656
Interventions required				
Reoperation turn to operating room	15 (18.1%)	34 (10.9%)	26 (10.3%)	0.029*
Angio-embolization	3 (3.6%)	7 (2.2%)	6 (2.4%)	0.095*
Hospital course				
Length of stay > 14 days	40 (48.2%)	132 (42.3%)	116 (46.0%)	0.584
LOS days, median (IQR)	20 (16.2–23.8)	17 (13.5–20.5)	19 (15.3–22.7)	-
ICU days, median (IQR)	4 (2.8–5.2)	3 (2.1–3.9)	3.5 (2.5–4.6)	-
CDc ≥ 3	33 (39.8%)	104 (33.3%)	57 (40.7%)	0.002†

*PBD* preoperative biliary drainage, *POVH* postoperative ventral hernia, *ARDS* acute respiratory distress syndrome, *MI* myocardial infarction, *AF* atrial fibrillation, *ICU* intensive care unit, *IQR* interquartile range, *CDc* Clavien-Dindo Classification

Significant p-values are indicated in bold

† *p* < 0.01 ** *p* < 0.05* ‡ Severe complications defined as Clavien-Dindo grade ≥ III

Long-term oncologic outcomes demonstrated variable patterns across treatment groups (Table 4). Disease-free interval and survival beyond 30 months showed no significant differences between groups (*p* = 0.073 and *p* = 0.578, respectively), although the median disease-free interval was notably shorter in high-bilirubin PBD patients (7 ± 1.4 months) compared to other cohorts (15–17 months). Mortality rates, however, exhibited significant stratification across all time points. Thirty-day mortality was significantly higher in high-bilirubin patients, regardless of drainage status (No PBD-High: 4.17% and the small excluded sample of PBD-High: 6.67%) compared to non-jaundiced patients (0.66–2.05%, *p* = 0.005). This disparity persisted at 90 days, with elevated mortality in No PBD-High (13.75%) versus 2.88–5.16% in other cohorts (*p* = 0.001). Similarly, overall morbidity demonstrated significant variation (*p* < 0.001), with the highest rates observed in No PBD-High (30.00%) group compared to 12.82% in No PBD-Low patients. Age categories did not influence the mortality rate.

**Table 4:** Patient outcomes by preoperative biliary status

Outcome measure	No PBD, high bilirubin (*n* = 83)	No PBD, low bilirubin (*n* = 312)	PBD group (*n* = 253)	*p*-value
Oncologic outcomes				
Disease-free interval				0.073
– Mean months (IQR)	17.0 (13.6–20.4)	16.0 (12.8–19.2)	15.5 (12.4–18.6)	
Survival > 30 months				0.578
– Events/evaluable patients	18/77 (23.4)	89/304 (29.3)	45/135 (33.3)	
– Mean months (IQR)	26.5 (22.3–30.7)	27.0 (22.7–31.3)	25.7 (21.5–29.8)	
Perioperative outcomes				
Overall morbidity	24/80 (30.0)	40/312 (12.8)	52/252 (20.6)	< 0.001***
Mortality				
30-day mortality	3/72 (4.2)	2/305 (0.7)	5/244 (2.1)	0.005**
60-day mortality	5/60 (8.3)	2/271 (0.7)	5/208 (2.4)	0.002**
90-day mortality	11/83 (13.3)	9/312 (2.9)	13/252 (5.2)	0.001***
Overall mortality	11/83 (13.3)	9/312 (2.9)	13/252 (5.2)	0.001***

Data are presented as n (%) or mean (interquartile range). High bilirubin defined as > 14.6 mg/dL; low bilirubin defined as < 14.6 mg/dL. Missing data ranges from 13.1% to 17.7% across outcomes

*PBD* preoperative biliary drainage, *IQR* interquartile range

^*^p < 0.05, **p < 0.01, ***p < 0.001

Detailed analysis of mortality in jaundiced patients without biliary drainage (Table 5). Within the high-bilirubin non-PBD cohort (*n* = 83), 11 patients (13.3%) died within 90 days postoperatively. This subset had a median age of 72 years (range 60–85), with a balanced gender distribution (6 males, 5 females). These patients exhibited distinctive morbidity patterns, with surgical site infections (63.6%), bleeding complications (54.5%), abdominal abscesses (45.5%), and ARDS (36.4%) occurring at substantially higher frequencies than in surviving patients. The median operative time was 6.7 h (range 4.7–8.6) with a median blood loss of 200mL (range 50–600 mL). Mortality was predominantly attributed to abdominal sepsis-related complications (63.6%), followed by respiratory failure/ARDS (36.4%), and pancreatic leak with sequelae (27.3%). Notably, these cases demonstrated significantly higher rates of ARDS and bleeding complications compared to other treatment groups (0.8–2.7% and 6.0–7.1%, respectively), with a median time to death of 36 days (range 10–80). These findings suggest that unrelieved severe jaundice may predispose patients to a cascade of inflammatory and infectious complications that significantly elevate early mortality risk.

**Table 5:** Characteristics and outcomes of patients with 90-day mortality after pancreaticoduodenectomy in the high-bilirubin non-PBD group

Case	Age/Sex	BMI (kg/m^2^)	ASA	Operative Time (h)	Blood Loss (mL)	Major Complications	ICU Stay (d)	Time to Death (d)	Primary Cause of Death
1	60M	25	II	6.0	200	SSI	3	16	Abdominal sepsis, pancreatic leak
2	68F	25	II	6.3	250	SSI, abdominal abscess	1	63	Abdominal sepsis
3	73M	32	III	5.9	100	SSI, ARDS, bleeding	3	41	Abdominal sepsis, early recurrence
4	75F	23	II	4.7	450	Abdominal abscess, bleeding	19	21	Abdominal sepsis, pancreatic leak
5	65F	28	III	6.7	600	SSI, abdominal abscess	2	10	Abdominal sepsis, deep wound infection
6	72F	33	III	7.8	200	ARDS, bleeding	1	36	Abdominal sepsis, ARDS
7	76F	29	III	7.8	400	SSI, abdominal abscess, bleeding	2	10	Abdominal sepsis, massive bleeding
8	70M	28	III	8.6	80	Abdominal abscess, ARDS, bleeding	2	80	Abdominal sepsis, pancreatic leak
9	85M	19	II	7.6	50	SSI, bacteremia	5	67	Abdominal sepsis
10	72F	33	III	7.8	200	ARDS, bleeding	1	36	Abdominal sepsis, ARDS
11	60M	25	II	6.0	200	SSI	3	16	Abdominal sepsis, pancreatic leak
Median	72	28	–	6.7	200	–	2	36	–
(Range)	(60–85)	(19–33)	–	(4.7–8.6)	(50–600)	–	(1–19)	(10–80)	–

This table details the characteristics and outcomes of 11 patients who died within 90 days after pancreaticoduodenectomy in the non-PBD high-bilirubin group (bilirubin ≥ 14 mg/dL). Infectious complications predominated with SSI (63.6%), abdominal abscess (45.5%), ARDS (36.4%), and bleeding (54.5%) events. The principal causes of death were abdominal sepsis-related

*ASA* American Society of Anesthesiologists. *BMI* body mass index. *SSI* surgical site infection. *ARDS* acute respiratory distress syndrome. *MOF* multi-organ failure. *DIC* disseminated intravascular coagulation. *PE* pulmonary embolism. *MI* myocardial infarction

Severe hyperbilirubinemia independently predicts mortality regardless of drainage status (Table 6). Multivariate logistic regression analysis identified preoperative bilirubi*n* > 14.6 mg/dL as the strongest independent predictor of 90-day mortality (OR 4.78, 95% CI 2.16–10.59, *p* < 0.001), superseding all other clinical variables. Postoperative pneumonia (OR 3.22, 95% CI 1.46–7.11, *p* = 0.004) and BMI > 30 kg/m^2^ (OR 2.84, 95% CI 1.15–7.02, *p* = 0.023) emerged as significant secondary predictors, while hypoalbuminemia (< 3g/dL) demonstrated a trend toward significance (OR 1.89, 95% CI 0.91–3.94, *p* = 0.089). Importantly, preoperative biliary drainage (*p* = 0.568), advanced age (*p* = 0.671), diabetes mellitus (*p* = 0.825), intraoperative blood loss (*p* = 0.222), packed cell transfusion (*p* = 0.948), and surgical site infection (*p* = 0.321) were not shown to be independent associations with mortality risk.

**Table 6:** Subgroup analysis of key parameters across biliary status groups

Parameter	No PBD, High Bilirubin (*n* = 83)	No PBD, Low Bilirubin (*n* = 312)	PBD- Low Bilirubin (*n* = 140)	PBD- High Bilirubin- Reduced (*N* = 113)	*p*-value
Demographics					
Age, mean (IQR)	69.5 (65.2–73.8)	64.1 (59.4–68.9)	68.0 (63.5–72.4)	67.1 (62.5–71.7)	0.074
Male, n (%)	55 (66.3)	166 (53.2)	71 (50.7)	76 (67.3)	0.011*
Albumin, mean (IQR)	38.8 (35.6–42.0)	40.2 (36.8–43.6)	38.9 (35.8–42.0)	35.7 (32.4–39.0)	< 0.001***
Clinical parameters					
Jaundice, n (%)	73 (96.1)	100 (32.4)	99 (71.7)	96 (99.0)	< 0.001***
Preop biopsy, n (%)	18 (30.5)	187 (65.6)	104 (82.5)	58 (69.9)	< 0.001***
Surgical outcomes					
Open approach, n (%)	48 (57.8)	58 (18.6)	41 (29.3)	40 (35.4)	< 0.001***
Blood loss > 500mL, n (%)	11/24 (45.8)	44/98 (44.9)	19/38 (50.0)	14/30 (46.7)	0.001**
Major complications					
SSI, n (%)	8 (9.6)	40 (12.8)	37 (26.4)	24 (21.2)	0.001***
ARDS, n (%)	5 (6.0)	4 (1.3)	2 (1.4)	0 (0)	0.016*
Reoperation, n (%)	15 (18.1)	34 (10.9)	13 (9.3)	13 (11.5)	0.029*
Outcomes					
Overall morbidity, n (%)	24/83 (29.0)	40/312 (12.8)	29/140 (20.7)	23/112 (20.5)	< 0.001***
90-day mortality, n (%)	11/83 (13.3)	9/312 (2.9)	8/140 (5.7)	5/112 (4.5)	0.001***

G1: No PBD with bilirubi*n* > 14.6 mg/dL; G2: No PBD with bilirubi*n* < 14.6 mg/dL; G3: PBD with initial bilirubi*n* < 14.6 mg/dL; G4: PBD with initial bilirubi*n* > 14.6 mg/dL reduced to < 14.6 mg/dL preoperatively; Statistical significance: *p < 0.05, **p < 0.01, ***p < 0.001

*SSI* Surgical site infection, *ARDS* Acute respiratory distress syndrome, *OR* Operating room, *PBD* Preoperative biliary drainage, *IQR* Interquartile range

Table 6 presents a comprehensive stratified analysis of clinically significant parameters across the four patient cohorts, revealing substantial differences in demographics, clinical presentation, surgical approach, complication profiles, and mortality outcomes.

Direct comparison between high-bilirubin patients with and without PBD (Supplemental Table S1) revealed significantly lower 90-day mortality (4.5% vs. 13.3%, *p* = 0.025), elimination of ARDS (0% vs. 6.0%, *p* = 0.007), and reduced severe complications (Clavien-Dindo ≥ 3: 26.5% vs. 39.8%, *p* = 0.050) in the PBD group, despite higher infectious complications (SSI 21.2% vs. 9.6%, *p* = 0.030). Table S2 shows the direct comparison between low- bilirubin patients with and without PBD.

## Discussion

Our study demonstrates an unacceptably high 90-day mortality (13.75%) and morbidity (30%) in patients with bilirubin levels exceeding 14.6 mg/dL who underwent direct pancreaticoduodenectomy without PBD. This investigation, spanning 17 years and including 83 patients with severe hyperbilirubinemia, represents the largest cohort studied for this specific high-risk group to date [18, 19]. Importantly, these differences persisted despite well-matched groups in terms of age (*p* = 0.074), comorbidities, and BMI (*p* = 0.412). Multivariate analysis confirmed severe hyperbilirubinemia as the strongest independent predictor of 90-day mortality (OR 4.78, 95% CI 2.16–10.59, *p* < 0.001), suggesting physiological derangements rather than patient selection drive these outcomes.

The management of severe jaundice remains controversial. Van der Gaag’s landmark RCT (2010) demonstrated increased complications with routine PBD [1] but excluded patients with bilirubi*n* > 14.6 mg/dL. Our larger cohort addresses this gap, supporting PBD’s role in severe hyperbilirubinemia.

The differential pathology distribution reflects periampullary tumor biology. Low-bilirubin non-PBD patients predominantly had ampullary adenocarcinomas and neuroendocrine tumors, presenting with earlier, milder jaundice due to distal anatomical location. Conversely, PDAC predominated in high-bilirubin groups (57.8%), causing severe obstruction through intrapancreatic bile duct compression. While PDAC prevalence differed between groups, the 90-day mortality endpoint reflects perioperative physiology rather than tumor biology, and the mortality benefit of PBD persisted when comparing groups with similar PDAC prevalence.

Clinicians frequently face patients whose bilirubin rises above 14.6 mg/dL during surgical preparation without evident cholangitis. Our four-group analysis provides crucial guidance: direct surgery with bilirubi*n* > 14.6 mg/dL yielded 13.3% mortality, while PBD-reduced patients achieved 4.5% mortality—comparable to low-bilirubin patients. This 66% relative risk reduction supports PBD when bilirubin exceeds 14.6 mg/dL, aligning with Shen et al. (2020) [19] and De Pastena et al. (2018) [22].

Severe hyperbilirubinemia’s impact stems from multiple mechanisms [11, 12]. Prolonged cholestasis impairs immune function, wound healing, and may induce hepatic fibrosis. Gdowski et al. (2017) demonstrated that undiagnosed liver fibrosis significantly affects pancreaticoduodenectomy outcomes [26]. Our higher complication rates in non-PBD high-bilirubin patients may reflect subclinical hepatic changes, representing another potential PBD benefit—allowing hepatic recovery before surgery. The lower biopsy rate in non-PBD patients (30.5% vs. 69.9–82.5%) reflects clinical urgency to proceed with surgery in unequivocal resectable disease. Patients proceeding directly to surgery typically presented with clear clinical and radiological evidence requiring expeditious intervention, while PBD patients had opportunities for tissue diagnosis during ERCP and additional time while awaiting bilirubin normalization [20, 24]. However, our mortality data suggests that PBD benefits may outweigh delays for tissue diagnosis even in these scenarios. The similar pancreatic duct diameters across groups suggest fistula differences resulted from hyperbilirubinemia’s physiological effects rather than anatomical variations.

While PBD increased infectious complications (SSI 24.2% vs. 9.6%, *p* = 0.001), aligning with Van der Gaag et al. [1, 7] and Chen et al. (2021) [23], these were primarily superficial infections manageable with antibiotics. Despite our prophylactic antibiotic protocol (piperacillin-tazobactam 24 h pre-ERCP, continuing 48–72 h), bacterial colonization remains challenging. However, the mortality benefit (13.3% vs. 4.5%) far outweighs these risks. The 18.1% reoperation rate in non-PBD high-bilirubin patients—driven by bleeding (54.5%) and abscesses (45.5%)—decreased to 9.3–11.5% with PBD (*p* = 0.029), suggesting improved tissue healing, though heightened surgical vigilance may also contribute.

The multivariate finding of hyperbilirubinemia as an independent predictor (OR 4.78, *p* < 0.001) doesn’t contradict PBD’s benefits. This paradox reflects residual hepatic dysfunction and cumulative jaundice effects. Notably, PBD-reduced patients achieved outcomes comparable to non-jaundiced patients, supporting drainage for bilirubin ≥ 14.6 mg/dL despite persistent risk, consistent with Dolejs et al. (2017) [25]. The striking mortality difference (13.3% vs. 2.9–5.2%) deserves scrutiny. While 2–5% mortality is standard for pancreaticoduodenectomy, outcomes for bilirubi*n* > 14.6 mg/dL without drainage are poorly studied. Our cohort represents one of the largest experiences. Table 5 shows mortality predominantly from abdominal sepsis (63.6%) and bleeding (54.5%), suggesting hyperbilirubinemia profoundly compromises immunity and hemostasis aligning with Swanson et al.’s (2014) findings [21].

Contemporary guidelines recommend selective PBD for severe jaundice [15, 17]. Our findings support extending this to patients whose bilirubin rises above 14.6 mg/dL during preparation. While only 13–14% of our patients received neoadjuvant therapy, current practice increasingly favors NAT, inherently selecting for PBD. Our data remains relevant for urgent surgery, non-PDAC pathologies, and resource-limited settings. The 90-day endpoint captures hyperbilirubinemia’s maximal physiological effects—immunity dysfunction, coagulopathy, impaired healing. Similar long-term survival (*p* = 0.578) confirms these effects are reversible and perioperative-specific. The higher proportion of open procedures in high-bilirubin non-PBD (57.8% vs. 18.6–32.0%) may reflect selection bias and temporal factors, but multivariate analysis confirmed surgical approach didn’t independently predict mortality.

Our retrospective, single-center design introduces potential bias. The 17-year span encompasses evolving techniques, though poor outcomes persisted throughout, suggesting hyperbilirubinemia’s impact transcends technological advances. Missing pancreatic duct data (32%) limited anatomical assessment. We lack comprehensive tumor biology markers (Ca19-9, resectability status), though these primarily affect long-term rather than 90-day outcomes. Despite transitioning from intermediate (40 cases/year) to high-volume status (55–70 cases/year), with fellowship-trained surgeons from high-volume centers, institutional volume evolution may influence results. Nevertheless, our mortality differences (9%) far exceed typical volume-related variations (3–5%). Surgeon experience may influence outcomes; however, our standardized protocols and experienced HPB team help minimize this variability.

## Conclusion

Preoperative bilirubin ≥ 14.6 mg/dL independently predicts increased mortality following pancreaticoduodenectomy. Despite infection risks, PBD’s mortality reduction from 13.3–4.5% supports drainage for severe hyperbilirubinemia. The 13.3% mortality precludes ethical randomization; future multicentre studies should validate findings and optimize drainage protocols.

## Supplementary Information

Below is the link to the electronic supplementary material.

Supplementary file1 (DOCX 18 kb)

## Abbreviations

ARDS: Acute respiratory distress syndrome
ASA: American Society of Anesthesiologists
BMI: Body mass index
CI: Confidence interval
DGE: Delayed gastric emptying
ERCP: Endoscopic retrograde cholangiopancreatography
HPB: Hepato-pancreato-biliary
ICU: Intensive care unit
IQR: Interquartile range
ISGPS: International Study Group of Pancreatic Surgery
LOS: Length of stay
LVI: Lympho-vascular invasion
MI: Myocardial infarction
MOF: Multi-organ failure
OR: Operating room
PBD: Preoperative biliary drainage
PDAC: Pancreatic ductal adenocarcinoma
PNI: Perineural invasion
PTD: Percutaneous transhepatic drainage
SSI: Surgical site infection
TNM: Tumor-Node-Metastasis
