# Supplementary material for: Impact of preoperative biliary drainage on outcomes of pancreaticoduodenectomy in severe hyperbilirubinemia
Source: Surg Endosc. 2025 Aug 13;39(10):6504–13. doi: 10.1007/s00464-025-12027-2 (PMC12500813; doi:10.1007/s00464-025-12027-2)
Supplement: Supplementary file 1 — Supplementary file1 (DOCX 18 kb) [file 464_2025_12027_MOESM1_ESM.docx]

**Table S1 (Supplemental): Direct Comparison of High-Bilirubin Patients With and Without Preoperative Biliary Drainage**

| **Parameter** | **No PBD-High Bilirubin (n=83)** | **PBD-High Bilirubin Reduced (n=113)** | **p-value** |
| --- | --- | --- | --- |
| **Demographics** |  |  |  |
| Age, mean (IQR) | 69.5 (65.2-73.8) | 67.1 (62.5-71.7) | 0.089 |
| Male sex, n (%) | 55 (66.3) | 76 (67.3) | 0.884 |
| Albumin, g/L, mean (IQR) | 38.8 (35.6-42.0) | 35.7 (32.4-39.0) | <0.001 |
| **Clinical Presentation** |  |  |  |
| Clinical jaundice, n (%) | 73 (96.1) | 96 (99.0) | 0.196 |
| Preoperative biopsy, n (%) | 18 (30.5) | 58 (69.9) | <0.001 |
| Preoperative bilirubin, mg/dL | 17.4 (14.6-27.7) | 4.3 (2.8-6.1)* | <0.001 |
| **Surgical Approach** |  |  |  |
| Open procedure, n (%) | 48 (57.8) | 40 (35.4) | 0.002 |
| Blood loss >500mL, n (%) | 11/24 (45.8) | 14/30 (46.7) | 0.948 |
| **Complications** |  |  |  |
| Surgical site infection, n (%) | 8 (9.6) | 24 (21.2) | 0.030 |
| Abdominal abscess, n (%) | 6 (7.2) | 17 (15.0) | 0.089 |
| Pancreatic fistula Grade B/C, n (%) | 14 (16.8) | 18 (15.9) | 0.866 |
| ARDS, n (%) | 5 (6.0) | 0 (0) | 0.007 |
| Pneumonia, n (%) | 7 (8.4) | 3 (2.7) | 0.066 |
| Reoperation, n (%) | 15 (18.1) | 13 (11.5) | 0.193 |
| **Primary Outcomes** |  |  |  |
| Overall morbidity, n (%) | 24 (29.0) | 23 (20.5) | 0.170 |
| 30-day mortality, n (%) | 3 (4.2) | 2 (1.8) | 0.329 |
| 90-day mortality, n (%) | 11 (13.3) | 5 (4.5) | 0.025 |
| Clavien-Dindo ≥3, n (%) | 33 (39.8) | 30 (26.5) | 0.050 |

*Post-drainage bilirubin level

**Note:** This direct comparison demonstrates the central finding of our study: preoperative biliary drainage in patients with severe hyperbilirubinemia (>14.6 mg/dL) significantly reduces 90-day mortality despite increased infectious complications.

**Table S2 (Supplemental): Direct Comparison of Low-Bilirubin Patients With and Without Preoperative Biliary Drainage**

| **Parameter** | **No PBD-Low Bilirubin (n=312)** | **PBD-Low Bilirubin (n=140)** | **p-value** |
| --- | --- | --- | --- |
| **Demographics** |  |  |  |
| Age, mean (IQR) | 64.1 (59.4-68.9) | 68.0 (63.5-72.4) | 0.002 |
| Male sex, n (%) | 166 (53.2) | 71 (50.7) | 0.625 |
| Albumin, g/L, mean (IQR) | 40.2 (36.8-43.6) | 38.9 (35.8-42.0) | 0.084 |
| **Clinical Presentation** |  |  |  |
| Clinical jaundice, n (%) | 100 (32.4) | 99 (71.7) | <0.001 |
| Preoperative biopsy, n (%) | 187 (65.6) | 104 (82.5) | <0.001 |
| Preoperative bilirubin, mg/dL | 4.2 (0.13-14.5) | 2.8 (0.2-13.5)* | 0.126 |
| **Surgical Approach** |  |  |  |
| Open procedure, n (%) | 58 (18.6) | 41 (29.3) | 0.010 |
| Blood loss >500mL, n (%) | 44/98 (44.9) | 19/38 (50.0) | 0.592 |
| **Complications** |  |  |  |
| Surgical site infection, n (%) | 40 (12.8) | 37 (26.4) | <0.001 |
| Abdominal abscess, n (%) | 27 (8.7) | 21 (15.0) | 0.042 |
| Pancreatic fistula Grade B/C, n (%) | 37 (11.9) | 24 (17.1) | 0.127 |
| ARDS, n (%) | 4 (1.3) | 2 (1.4) | 0.895 |
| Pneumonia, n (%) | 17 (5.4) | 3 (2.1) | 0.109 |
| Reoperation, n (%) | 34 (10.9) | 13 (9.3) | 0.597 |
| **Primary Outcomes** |  |  |  |
| Overall morbidity, n (%) | 40 (12.8) | 29 (20.7) | 0.030 |
| 30-day mortality, n (%) | 2 (0.7) | 3 (2.1) | 0.150 |
| 90-day mortality, n (%) | 9 (2.9) | 8 (5.7) | 0.137 |
| Clavien-Dindo ≥3, n (%) | 104 (33.3) | 57 (40.7) | 0.129 |

*Post-drainage bilirubin level
